# Development and Application of a Liquid Chromatography–Tandem Mass Spectrometry Method for the Analysis of 20 Perfluoroalkyl Substances in Fruit and Vegetables at Sub-Parts-per-Trillion Levels

**DOI:** 10.1021/acs.jafc.4c01172

**Published:** 2024-08-07

**Authors:** Ruben Kause, Stefan van Leeuwen, Kerstin Krätschmer, Bob van Dooren, Rens Keppels, Helgah Makarem, L. Ron A. P. Hoogenboom, Leontien de Pagter-de Witte, Bjorn J. A. Berendsen

**Affiliations:** Wageningen Food Safety Research (WFSR), Wageningen University & Research, 6708 WB Wageningen, Netherlands

**Keywords:** PFASs, LC−MS/MS, food, validation, exposure

## Abstract

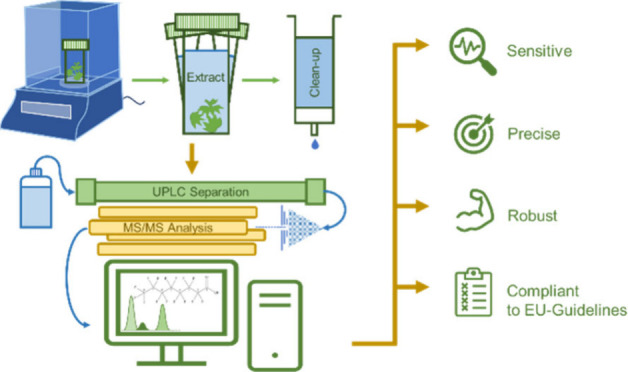

In response to the European Food Safety Authority’s establishment of a tolerable weekly intake (TWI) for the sum of PFOA, PFNA, PFHxS, and PFOS, a method was developed to quantify and confirm 20 PFASs at the sub-parts-per-trillion level in fruit and vegetables. Improved sensitivity was achieved by (i) increasing the sample intake, (ii) decreasing the solvent volume in the final extract, and (iii) using a highly sensitive mass spectrometer. Except for PFTrDA, target PFASs could be quantitatively determined with an apparent recovery of 90–119%, limits of quantitation down to 0.5 ng/kg, and a relative standard deviation under within-laboratory reproducibility conditions of <28%. The method was successfully applied to 215 fruit and vegetable samples obtained from local grocery stores and markets. Leafy vegetables prove to be the main vegetable category responsible to PFAS exposure, mainly of PFOA, followed by PFHpA and PFHxA.

## Introduction

Per- and polyfluoroalkyl substances (PFASs) are an extensive class of synthetic chemicals known for their chemical and heat resistance as well as their ability to strongly reduce surface tension. They have been extensively manufactured and utilized in various industries due to these desirable properties.^1^ They have been used in the production of non-stick cookware, waterproof clothing, and fire-fighting foams, among other products.

Due to increasing global concern about the potential negative health effects of PFASs, the European Food Safety Authority (EFSA) conducted a new risk assessment of PFASs in food. EFSA derived a tolerable weekly intake (TWI) of 4.4 ng/kg of body weight per week for the sum of four PFASs. These PFASs are perfluorooctanoic acid (PFOA), perfluorononanoic acid (PFNA), perfluorohexanesulfonic acid (PFHxS), and perfluorooctanesulfonic acid (PFOS); the so-called “EFSA-4”. It was shown that current exposure of a large part of the European Union (EU) population exceeds this TWI, even when applying the lower-bound principle (i.e., assuming that non-detected levels are equal to zero). The upper-bound exposure [i.e., assuming that non-detected levels equal the concentration of the limit of quantification (LOQ)] was much higher, implying a large uncertainty in the assessment and the need to apply more sensitive analytical methods.

The stringent requirements of the low TWI necessitate the use of highly sensitive analytical methods with low limits of quantification. When methods with relatively high LOQs are used, the majority of analyses yield non-detectable results. Typically, exposure assessments are conducted under an upper-bound scenario, where samples with non-detects are assumed to contain PFASs at the LOQ. Following this principle, if the method’s LOQs are too high, PFAS exposure can surpass the new TWI by many orders of magnitude, even in the absence of detected PFASs in the samples.

Considering the potential harm associated with PFASs, even at low concentrations, there is an urgency to develop analytical methods with low detection limits for various food products, as emphasized by EFSA.^2^ The European Union Reference Laboratory for Persistent Organic Pollutants in Feed and Food (EURL-POPs) has issued guidance on PFAS analysis, specifying that for fruit and vegetables, LOQs should be ≤5 ng/kg for PFNA, ≤10 ng/kg for PFOA and PFOS, and ≤15 ng/kg for PFHxS.^3^ Furthermore, laboratories are encouraged to aim for even lower LOQs, specifically ≤1 ng/kg for PFOA and PFNA, ≤2 ng/kg for PFOS, and ≤4 ng/kg for PFHxS. These latter LOQs have been adopted by Commission Regulation EU 2022/1431 as mandatory for monitoring purposes.^4^

Within the food domain, according to EFSA,^2^ fish and other seafood are the main sources of exposure to PFOA and PFOS, followed by eggs, meat products, and fruit. Notably, fruit and vegetables are an important source of exposure to PFOA, because of their substantial consumption compared to other foods.

Recent literature reviews have explored analytical methodologies for PFAS analysis and their occurrence in various sources, including food^5,6^ Furthermore, in recent years, there has been an expanded focus on examining the presence and transfer of PFASs in fruit and vegetables. Various studies describe methodologies to monitor PFAS levels in fruit and vegetables.^7−20^ Additionally, some studies have documented analytical methods to study the transfer of several PFASs from contaminated irrigation water to crops.^21−23^ Regrettably, most of the developed methods did not meet the targeted and/or proposed LOQs currently required by the EURL-POPs^3^ and commission regulation EU 2022/1431.^4^ Most methods were validated at relatively high concentration levels, and/or no fit-for-purpose validation was reported. Table 1 offers a comparison of recent studies on the analysis of PFASs in fruit, vegetables, and other plant material, highlighting the substantial variability in analytical characteristics of current methods. As a result, only scarce high-quality quantitative data on PFASs in vegetables at required concentration levels was available prior to the study presented here.

**Table 1:** Comparison of Different Methods for PFAS Analysis in Fruit, Vegetables, and Other Crops

						detection/quantification limit		
authors	matrix	extraction method	clean-up procedure	instrumentation	targeted PFAS	value	methodology	lowest recovered spike level
Zhou et al.^24^	vegetables	acetonitrile + formic acid	Sin-QuEChERS (PSA, C18, and GCB)	UHPLC–MS/MS	20 PFASs, including PFCAs, and PFSAs	0.003–0.034 μg/kg (LOQ)	10× S/N	0.1 μg/kg
Li et al.^9^	vegetables	methanol	online SPE^a^	UHPLC–MS/MS	21 PFASs, including PFCAs, PFSAs, and FTSs^b^	0.002–0.008 μg/kg (LOD)	3× SD of spike (0.2 μg/kg)	0.2 μg/kg
Nassazzi et al.^25^	plant material	methanol	ENVI carb cartridge	UHPLC–MS/MS	24 PFASs, including PFCAs, PFSAs, FASAs,^c^ and FTSs	0.01–11.0 μg/kg (LOQ)	10× S/N	0.025 μg/kg
Meng et al.^7^	fruit and vegetables	methanol + ammonium hydroxide	WAX SPE	UHPLC–MS/MS	45 PFASs, including PFCAs, PFSAs, PFEAs,^d^ FASAs, FTSs, FTCAs,^e^ and PFESAs^f^	0.025 to 0.25 ng/g (LOQ)^g^	lowest recovered solvent standard × matrix effect	1 μg/kg
Piva et al.^26^	vegetables	acetonitrile + formic acid	WAX SPE	UHPLC–MS/MS	22 PFASs, including 3 FTSs	0.05–0.5 μg/kg (LOQ)	10× S/N	1 μg/kg
Zacs et al.^27^	fruit and vegetables	acetonitrile + NaOH	WAX SPE	nano-LC–nano-ESI–Orbitrap MS	EFSA-4	0.001–0.002 μg/kg	lowest validated spike	0.001 μg/kg^h^

a Sorbent was not provided in the publication.

b FTSs = fluorotelomer sulfonates.

c FASAs = polyfluoroalkyl sulfonamides.

d PFEAs = polyfluoroalkyl Ether Acids.

e FTCAs = fluorotelomer carboxylic acids.

f PFESAs = perfluoroalkyl ether sulfonic acids.

g The LOQ was determined as the lowest calibration level that could accurately be determined in solvent, corrected by the calculated matrix effects to yield estimated LOQs in food sample extracts.

h PFOA was spiked at 0.002 μg/kg to overcome PFOA backgrounds in procedural blanks.

In the current study, a method was developed and validated to detect and quantify 20 PFASs, including PFOA, PFNA, PFHxS, and PFOS, at the low ppt (ng/kg) level in a wide range of fruit and vegetables (see SI-2 of the Supporting Information). This study is the first description of a method that can achieve the very low detection limits required for human exposure assessments of PFAS via fruit and vegetables. The achievement of such low detection limits is especially challenging as background contamination of commonly applied PFAS becomes apparent. Also, we demonstrate an extensive validation protocol to include a wide range of vegetables. The method was subsequently applied to a selection of fruit and vegetables obtained from local grocery stores and weekly markets (*n* = 215).

## Materials and Methods

### Chemicals

Methanol (MeOH) and acetonitrile of UHPLC/MS grade were purchased from Actu-All Chemicals (Oss, Netherlands). UHPLC/MS grade water was procured from Biosolve (Valkenswaard, Netherlands). All other chemicals were obtained from Merck (Darmstadt, Germany). A 2% ammonium hydroxide solution was prepared by diluting a 25% ammonium solution 12.5 times in acetonitrile. A 25 mM sodium acetate buffer was prepared by dissolving 3.40 g of sodium acetate trihydrate in 1 L of water and adjusting to pH 4 with glacial acetic acid. A 4 M hydrochloric acid solution was prepared by diluting 3.3 mL of 37% HCl to 10 mL with water, and lower concentrations were prepared by diluting this solution. Mobile phase A was a 20 mM ammonium acetate in water solution, was prepared by dissolving 1.54 g of ammonium acetate in 1 L of water. Mobile phase B was methanol.

### Reference Standards

All reference standards were obtained from Wellington Laboratories (Guelph, Ontario, Canada). The following perfluoroalkyl carboxylic acids (PFCAs) were used in this study: perfluoropentanoic acid (PFPeA, C_5_), perfluorohexanoic acid (PFHxA, C_6_), perfluoroheptanoic acid (PFHpA, C_7_), PFOA (C_8_), PFNA (C_9_), perfluorodecanoic acid (PFDA, C_10_), perfluoroundecanoic acid (PFUnDA, C_11_), perfluorododecanoic acid (PFDoDA, C_12_), perfluorotridecanoic acid (PFTrDA, C_13_), and perfluorotetradecanoic acid (PFTeDA, C_14_). All PFCAs were obtained as a mixture of 2 μg/mL in MeOH.

The following perfluoroalkyl sulfonic acids (PFSAs) were used in this study: perfluorobutanesulfonic acid (PFBS, C_4_), PFHxS (C_6_), perfluoroheptanesulfonic acid (PFHpS, C_7_), PFOS (C_8_), and perfluorodecanesulfonic acid (PFDS, C_10_). These PFSAs were obtained as individual solutions of their sodium salts (except PFBS, which is a potassium salt) of 2 μg/mL in MeOH. Additionally, a few other PFASs were included in this study. Those being: perfluorooctanesulfonamide (PFOSA), hexafluoropropylene oxide–dimer acid (HFPO–DA), also known as GenX technology, Sodium dodecafluoro-3*H*-4,8-dioxanonanoate (NaDONA), sodium dodecafluoro-3*H*-4,8-dioxanonanoate (9Cl-PF3ONS), and sodium dodecafluoro-3*H*-4,8-dioxanonanoate (11Cl-PF3OUdS). These compounds were also obtained at a concentration of 2 μg/mL in MeOH. All reference compounds have a chemical purity of at least 98%.

Isotopically labeled compounds were used as internal standards in this study. A mixture containing the following compounds was obtained at a concentration of 2 μg/mL in methanol: ^13^C_2_-PFHxA, ^13^C_4_-PFOA, ^13^C_5_-PFNA, ^13^C_2_-PFDA, ^13^C_2_-PFUnDA, ^13^C_2_-PFDoDA, ^18^O_2_-PFHxS, and ^13^C_4_-PFOS. Additionally, ^13^C_3_-PFPeA, ^13^C_4_-PFHpA, ^13^C_3_-PFBS, and ^13^C_3_-HFPO–DA were obtained as individual solutions at the same concentration. Isotopically labeled ^13^C_8_-PFOA and ^13^C_8_-PFOS standards were used as injection checks (2 μg/mL). All labeled compounds had a chemical purity of at least 98% and isotopic purities of at least 99% for ^13^C and 94% for ^18^O.

### Sample Preparation

Ten grams of sample were transferred to a 50 mL polypropylene (PP) centrifuge tube (Greiner Bio-One, Kremsmünster, Austria). The sample was then fortified with 50 μL of internal standard solution (1 ng/mL) and 0.5 mL of 200 mM sodium hydroxide solution was added, followed by 10 mL of MeOH. The mixture was vortexed for 1 min in a multivortex mixer (VWR, VX-2500 Vulti-Tube Vortexer, Radnor, PA, U.S.A.), followed by 15 min of ultrasonication at room temperature (in an ultrasonic bath by Branson, Danbury, CT, U.S.A.) and 30 min of shaking on a rotary tumbler (REAX-2, Heidolph, Schwabach, Germany). After the extraction, 100 μL of formic acid was added and the mixture was centrifuged for 10 min at 3600 rpm at 10 °C (Rotixa 500 RS, Hettich Zentrifugen, Westphalia, Germany). The supernatant was then carefully decanted into a 50 mL PP tube that contained 25 mL of HPLC-grade water. The extract was mixed and centrifuged again if cloudy, before the cleanup.

For cleanup and further concentration of the sample, a Strata-X-AW cartridge (mixed mode weak anion exchange, 200 mg per 6 mL, 33 μm; Phenomenex, Torrance, CA, U.S.A.) was conditioned with 8 mL MeOH and then 8 mL of 0.04 M HCl. The extract was transferred onto the cartridge and slowly passed through (if necessary, by applying a vacuum) to allow interaction between the SPE material and the PFASs. The cartridge was then rinsed with 5 mL of 25 mM sodium acetate buffer, followed by 3 mL of 0.04 M HCl in MeOH. The PFASs were eluted from the cartridge using 5 mL of 2% ammonium hydroxide in acetonitrile and collected into a 14 mL PP tube (Greiner Bio-One, Kremsmünster, Austria).

The solvent was evaporated (at 40 °C using nitrogen gas) using a TurboVap LV Evaporator (Zymark, Hopkinton, MA, U.S.A.). After evaporation to dryness, 80 μL MeOH, 270 μL ammonium acetate buffer (20 mM), and 50 μL of the injection standard mixture (1 ng/mL) (containing ^13^C_8_-PFOA and ^13^C_8_-PFOS) were added. The residues were then reconstituted by rigorous mixing (vortex mixer) and 5 min of ultrasonication. The final extract was passed through a 0.45 μm regenerated cellulose syringe filter (Whatman, Little Chalfont, Buckinghamshire, U.K.) before LC–MS/MS analysis.

### UPLC–MS/MS

The UPLC–MS/MS analysis was performed using a Sciex ExionLC UPLC system (Sciex, Framingham, MA, U.S.A.). A Luna Omega PS C18 analytical column (100 Å, 100 × 2.1 mm inner diameter, 1.6 μm, Phenomenex, Torrance, CA, U.S.A.) was used to separate the PFASs at a column temperature of 40 °C. Additionally, a Gemini C18 analytical column (110 Å, 50 × 3 mm inner diameter, 3 μm, Phenomenex) was used as an isolator column, placed between the pump and the injector valve to isolate and delay potential PFAS contamination eluting from the LC system parts prior to the injection valve. The gradient: 0–1.5 min, 20% mobile phase B, 1.5–9.5 min, linear increase to 98% B with a final hold of 1.4 min. The gradient was returned to its initial conditions within 0.1 min and the column was allowed to equilibrate for 2.5 min before the next injection was initiated, resulting in a total run of 13.5 min. The flow rate was 0.5 mL/min and the injection volume 20 μL.

The detection of PFASs was done using MS/MS on a Sciex QTRAP 7500 system in negative electrospray ionization (ESI−) mode. The ion spray voltage, curtain gas, source temperature, gas 1, gas 2, and collision gas were set at −1500 V, 45 psi, 400 °C, 40, 80, and 9 psi, respectively. To fragment the PFASs, collision-induced dissociation (CID) was used with argon as the collision gas. The analysis was performed in multiple reaction monitoring (MRM) mode, using two mass transitions per component (except for PFPeA), which were selected based on the abundance of the signal and the selectivity of the transition. Additional information on the MRM transitions, entrance potential, collision energy, and cell exit potential can be found in Table S1 of the Supporting Information. The data were acquired using SciexOS and processed using MultiQuant software (SCIEX, Framingham, MA, U.S.A.).

### Blank Level Management

To reduce the risk of contamination through material selection, all fluoropolymer containers (tubes, vials, etc.) and devices (filters, pipets, etc.) were excluded from the method, if possible. The analysts did not wear any cosmetics or PFAS-containing clothing during sample handling, as required by EU regulations (EU) 2022/1428. Although no significant concentrations of PFASs were observed in the procedural blanks, except for PFBA, PFPeA, and small amounts of PFOA, it is recommended to test solvents and chemicals for PFASs prior to method development. Several sources with low-contamination were identified and eliminated. This test became essential only after the need for very low detection limits.

To test and correct for incidental contamination originating from the laboratory or laboratory consumables, blank chemical preparations (procedural blanks) were carried out in duplicate each day. The signal of all samples was corrected with the average response of the procedural blanks. The impact of interfering signals becomes more pronounced with extremely low method detection limits.

### Validation

This validation study aimed to cover a broad range of commonly consumed fruit and vegetables in the Netherlands. As a basis for the validation of the EURL POPs guidance document on PFAS analysis in food^3^ was applied. The validation was done more extensively than required by that document.

The following parameters related to a quantitative confirmatory method were determined: selectivity, stability, robustness, apparent recovery (trueness based on spiked samples), within-laboratory reproducibility (expressed as relative standard deviation, RSD_RL_), repeatability (expressed as relative standard deviation, RSD_r_), limit of detection (LOD), limit of quantification (LOQ), and limit of confirmation (LOC).

#### Validation Design

The method was characterized as a quantitative confirmatory method and the validation was designed to challenge fit-for-purpose for this goal. Fruit and vegetables were selected and subdivided into five matrix categories: leafy vegetables, fruit, root vegetables, bulb vegetables, and leek, and “other vegetables” mostly containing fruiting vegetables, legumes, and cabbage. The validation of each matrix category was performed on a single day, yielding a total of 5 validation days (Table 2). For each category, a representative matrix was selected for preparation of the matrix-fortified calibration (P1, Table 2). Furthermore, an additional six matrices (P2–P7, Table 2) of each category were analyzed as is (blank) and with the addition of all 20 PFASs at 2.5, 50, and 500 ng/kg. A detailed overview of the validation design is given in Table S2 of the Supporting Information.

**Table 2:** Selected Samples for the Validation Study^a^

number	leafy vegetables	bulb vegetables and leek	root vegetables	fruit	other vegetables
P1 (MFS)^b^	spinach	onion	potato	apple	zucchini
P2	endive	onion	beets (peeled)	strawberry	cauliflower
P3	kale	leek	beets (unpeeled)	white grape	broccoli
P4	iceberg lettuce	garlic	carrot (peeled)	plum	snow peas
P5	Turkish lettuce	red onion	carrot (unpeeled)	pear	rhubarb
P6	chard	scallions	potato (peeled)	red berries	pumpkin
P7	Batavia lettuce	chives	potato (unpeeled)	apple	cucumber

a The samples were divided into five matrix categories with 6 matrices each (excluding the calibration matrix).

b MFS = matrix-fortified standard.

#### Quantification

One specific sample batch (P1, Table 2) was selected for matrix-fortified standard calibration on each day. The matrix fortified standards (MFS calibration standards) included the following concentration levels to cover a wide concentration range: 0, 0.5, 1.0, 2.5, 5.0, 10, 25, 50, 100, 500, 1000, and 2000 ng/kg. Based on the PFAS concentration in the samples, the lower or higher end of the calibration line was used for quantification. Quantitative results were achieved using the matrix-fortified standard calibration approach, which involved correcting the signals (peak area) of the individual PFASs with the corresponding isotopically labeled internal standards. This correction accounts for differences in the recovery, ionization, and other matrix influences. For PFTrDA, PFHpS, PFDS, DONA, 9Cl-PF3ONS, and 11Cl-PF3OUdS no labeled internal standard were available. For these compounds, an internal standard was selected based on their retention time and chemical similarities. Retention time was the most important factor. The internal standards used per analyte are included in Table S1 of the Supporting Information.

#### Confirmation of Peak Identity

For confirmatory analysis, criteria have been established in the EURL-POPs guidance document^3^ for the maximum allowed deviation of the relative abundance of both diagnostic ions (ion ratio) resulting from an unknown sample. The maximum allowed deviation is 30%. Furthermore, the relative retention time of a PFAS should not deviate more than 1% from the reference relative retention time. To assess the possibility of confirming the identity of a detected compound using the presented method the average ion ratio and the average relative retention time of the matrix-fortified standard calibration samples was used as the reference value.

#### Selectivity, Stability, and Robustness

The EURL-POPs guidance document^3^ states that analytical methods should demonstrate the ability to reliably and consistently separate the analytes of interest from other coextracted and possibly interfering compounds that may be present. It is known that PFOS detection may suffer from a coeluting interference of taurodeoxycholic acid (TDCA), which is a bile acid with the same transition as the most sensitive PFOS transition (*m*/*z* 499 > 80).^28^ This bile acid is particularly prominent in eggs.^29^ In this method TDCA was chromatographically separated from PFOS and the mass transition *m*/*z* 499 > 99 was applied for quantitative purposes, preventing any interference. Moreover, it is unlikely that this bile acid interference occurs in fruit and vegetables. Additionally, it is noteworthy that although the *m*/*z* 499 to 99 transition is 75% less sensitive, it offers much greater specificity, resulting in fewer observed interferences in general. The robustness of the method was challenged by including many different fruit and vegetables. Furthermore, the validation was carried out on five different days and by three different technicians.

The stability of the PFASs in the samples and solvent solutions was not tested as it is generally agreed upon that these substances are very persistent. From the PFASs included in this study, only HFPO–DA is known to degrade to heptafluoropropyl 1,2,2,2-tetrafluoroethyl ether in aprotic polar solvents, such as dimethyl sulfoxide, acetone, and to a lesser extent in acetonitrile; with 100% degradation after approximately 15 h.^30,31^

Additionally, Zhang et al. showed that the degradation of HFPO–DA in acetonitrile was negligible in the presence of water (>20%), suggesting that acetonitrile can be used as a solvent for sample preparation when the water content is >20%.^31^ In our experiments, the lowest water concentration in acetonitrile of the extract is approximately 8%, under alkaline conditions. Under these conditions, we therefore assume that the degradation of HFPO–DA is limited, but not excluded. To test this hypothesis, a single-factor ANOVA was performed on the relative standard deviation of the signal of the internal standards for HFPO-DA, PFOA, and PFOS; two PFASs that are considered to be very persistent. We assume that there would be a larger variance in signal intensity of HFPO-DA, when degradation is a critical factor.

#### Apparent Recovery (Trueness), Repeatability, and Within-Laboratory Reproducibility

For the calculation of apparent recovery (trueness), repeatability, and within-laboratory reproducibility for each PFAS the quantitative data obtained from the samples spiked at 2.5, 50, and 500 ng/kg of each analyte was used. The apparent recovery for each sample was calculated by dividing the calculated concentration by the actual spiked concentration, in some cases after correction for a signal found in the procedural blank or the non-fortified sample. The reported apparent recovery for a specific PFAS is the average of all spiked samples at a concentration level. The relative standard deviation under repeatability conditions (RSD_r_) was calculated from all the individual analyzed matrices within a single matrix category for each concentration level. The relative standard deviation under within-laboratory reproducibility conditions (RSD_RL_) was calculated from all matrices at each concentration level. Note that in this validation design, for repeatability calculations different matrices are included. Therefore, the result is an overestimation of the actual repeatability. This approach was used to determine the overall performance of the method with a very high variation in types of fruit and vegetables.

The performance criteria were established in advance and derived from the EURL POPs guidance document.^3^ The guidance document differentiates analysis for compliance testing and analysis for monitoring purposes. Compliance testing relates to the EFSA-4 PFASs at the regulatory level. As for fruit and vegetables, no regulatory limits have been established, in this validation the method performance criteria for monitoring apply. The apparent recovery must lie between 65 and 135%, RSD_RL_ should be ≤25%. No criterion for RDS_r_ is established.

#### Limit of Detection, Quantification, and Confirmation

As this method would be applied to food exposure studies, it is crucial to establish limits for determining the absence and presence of specific substances. To accomplish this, we have adopted the approach previously described by Berendsen et al.,^32^ with a focus on the LOQ and LOC.

The LOQ represents the concentration at which a quantitative result can be obtained, typically based on a single ion transition, whereas confirmation of the identity at this concentration may not be possible. Concentrations at or below the LOQ are used to report the absence of a substance, based on this single ion transition. The LOC is considered to be the lowest concentration level of a PFAS at which it complies with the confirmatory criteria, as described under “Confirmation of Peak Identity”.^33^

For some substances, signals in the procedural blanks, originating from e.g. solvents, are common. Therefore, we applied two different strategies to determine the LOQ and LOC. One approach is employed when no substantial signal is observed in the procedural blanks, while the other is used when a substantial signal is detected in the procedural blanks.

If no signal of a specific PFAS is detected in the procedural blank samples, we established the LOQ as the lowest spiked concentration in the MFS calibration line with a signal-to-noise ratio ≥6.^34^ The LOC, in this case, is defined as the lowest spiked concentration in the MFS calibration line, meeting the confirmatory requirements.

On the other hand, when a signal is detected in the procedural blank, we follow the guidelines set by the EURL,^3^ which provides that the contribution of blank levels should not exceed 30% of the levels in the samples analyzed in the accompanying batch. In such a case, the LOQ was determined by multiplying the concentration of the PFAS in the procedural blank by a factor of 3.3. The LOC remains as the lowest spiked concentration in the MFS calibration line that meets the confirmatory requirements. If, in this scenario, the determined LOC is lower than the LOQ, it is set equal to the LOQ. In any case, the determined LOQ and LOC are assessed by comparing them to the results of the spiked validation samples and adjusted accordingly if needed (e.g., in case the LOQ derived from the MFS seems unachievable or unrealistic as that is only derived from a single matrix).

### Application

The developed method was applied to the analysis of 215 fruit and vegetables obtained from Dutch grocery stores and weekly markets, of which 35 leafy vegetables, 25 root vegetables, 23 bulb vegetables and leek, 50 fruit, and 82 other vegetables. The samples were collected and analyzed in 2021. A list of samples and their land of origin is included in SI-4 of the Supporting Information.

## Results and Discussion

### Method Development

Not all substances recommended by the EURL-POPs were included in this study, such as some long-chain PFSAs (perfluoroalkyl sulfonic acids) and next-generation PFASs.^3^ These compounds were at the time unavailable to the laboratory.

Achieving the required LOQs for fruit and vegetables poses a significant challenge due to their exceptionally low target thresholds and diverse matrices. Our strategy to achieve the lowest possible LOQs involves increasing the concentration factor of samples by increasing the sample intake and lowering the extract reconstitution volume. However, practical constraints, such as the capacities of extraction tubes, shaking equipment, and centrifuges, limit the sample intake volume.

It is crucial to fine-tune the extraction process as well, focusing on optimizing the solvent and solvent-to-sample ratio to allow the extract to run through the solid-phase extraction (SPE) cartridge. In the case of certain fruit and vegetables, the final extracts exhibited turbidity. A filtering step was therefore a requirement. Even after filtering, some extracts were somewhat turbid, demonstrating that the practical limitations of the method had been reached. Final extracts that were still turbid were shortly centrifuged, using a high-speed centrifuge, at 12 000 rpm.

The extraction process presented particular challenges when dealing with leafy greens, as they tended to yield cloudy extracts. Moreover, the preparation of certain leafy greens, like chives and leek, occasionally proved cumbersome, especially during the grinding process, due to their unique textures and structures. The fibrous nature and large, flat surface areas of some leaves made grinding a labor-intensive task. These combined factors contribute to the complexity of the analytical process in this study.

To gain insights into the performance of the analytical process, we introduced internal standards into the samples prior to the preparation stage and added injection standards just before the sample injection. The injection standard consists of two isotopically labeled analogs of PFOS and PFOA (see SI-1 of the Supporting Information). Assessing the relative abundance of the internal- and injection standards, we found absolute recoveries ranging between 41 and 79% for PFOA and 32 and 63% for PFOS. Notably, bulb vegetables exhibited substantially lower absolute recoveries (32–53%) compared to other fruit and vegetables. Matrix effects for PFOA and PFOS were determined by comparing the injection standard added to sample matrices after cleanup with the injection standard added to the procedural blank, revealing a range from 32% for bulb vegetables to 157% for leafy vegetables. The matrix effect could only be determined for PFOA and PFOS since isotopically labeled variants (^13^C_8_) of those PFAS were included in the injection standard.

The hydrophobic nature of the PFASs included in this study is very diverse, as indicated by the octanol–water partitioning coefficient (*K*_ow_) ranging from 3.4 for PFPeA to 7.15 for PFUnDA,^35^ with higher values for the longer chain PFASs (no data available).^36^ Prior work by Zenobio et al. highlighted the adsorption of hydrophobic PFASs to container surfaces.^36^ From the recovery experiments in the current study, this effect was observed for the long-chain PFASs (≥C_12_). Approximate 50% MeOH is required to keep these PFASs in solution in the glass LC vial. However, a high organic solvent percentage in the final extract jeopardizes the chromatographic separation of short-chain PFAS. To address this, we opted for a final extract composition containing 32.5% MeOH, ensuring satisfactory peak shapes for the early eluting PFASs and an acceptable recovery for the long-chain PFASs.

**Figure 1:**
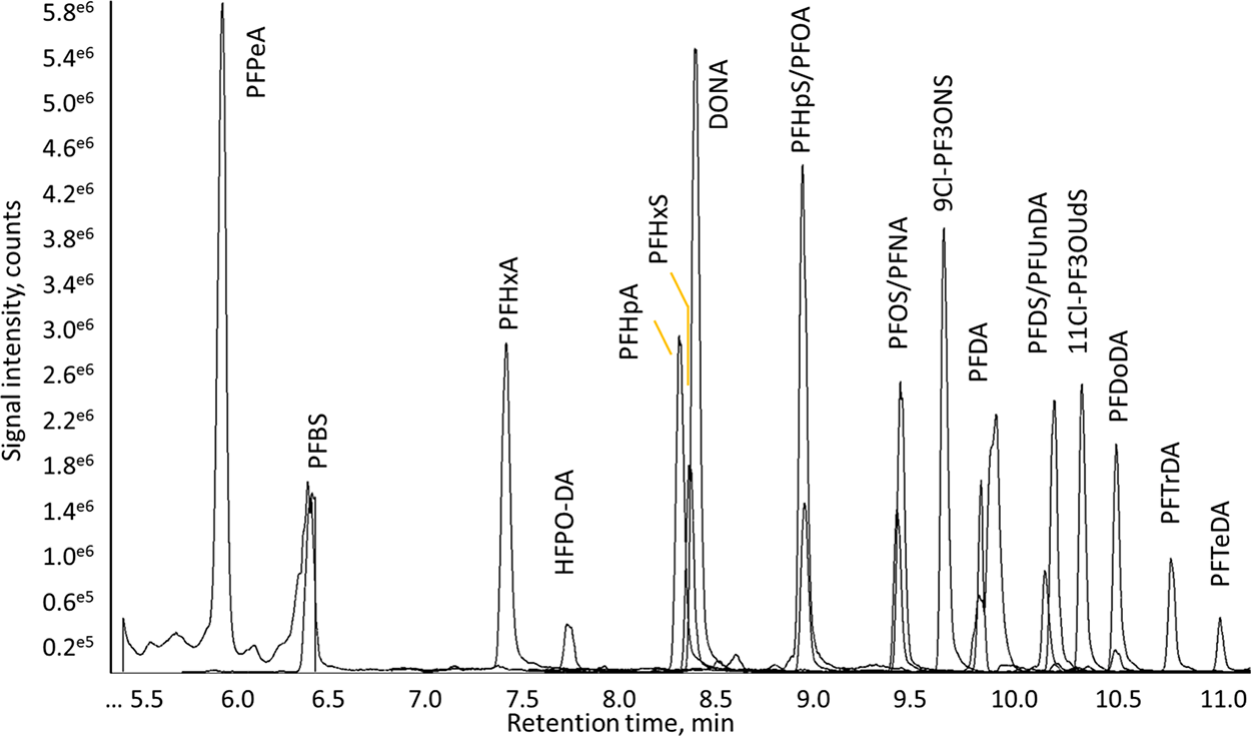
MRM chromatogram of all 20 PFASs in a spiked potato sample at 10 ng/kg. PFOSA is not visible in the current view but elutes after 10 minutes.

Given that long-chain PFASs (≥C_12_) were anticipated to be present in crops to a lesser extent than shorter chain PFASs,^37^ an absolute recovery within the range of approximately 5 to 20% compared to PFOA was deemed an acceptable threshold. PFHxDA (perfluorohexadecanoic acid) and PFODA (perfluorooctadecanoic acid) were originally included in the method development. However, it demonstrated extremely low absolute recovery under the current conditions. Given the unlikely accumulation of these compounds in fruit or vegetables, we adjusted the method’s focus toward more hydrophilic PFASs. During method development and validation, perfluorobutyric acid (PFBA) was also considered. Unfortunately, it displayed severe background signals in all injections, restricting the method’s applicability (see SI-3 of the Supporting Information). Consequently, PFBA was excluded from the method. A MRM chromatogram of a potato sample spiked at 10 ng/kg with all 20 PFASs is presented in Figure 1. In SI-3 of the Supporting Information, example chromatograms are included of unspiked samples, and at 1 ng/kg.

In examining the selectivity challenges posed by both PFBA and PFPeA, which have only a single sufficiently abundant product ion in MS/MS detection, the method’s limitation becomes apparent. It becomes difficult to conclusively determine whether an observed signal is related to the presence of an interfering substance or if PFBA or PFPeA is genuinely present in the chromatogram. The few publications that integrated PFBA and PFPeA in their methods and reported their presence in fruit and vegetables share this limitation, often without addressing the lack of selectivity. Therefore, findings related to PFBA and PFPeA should be interpreted with caution. To address this selectivity issue, we introduced the ion transition from precursor ion mass to precursor ion mass at low collision energy for PFPeA, allowing for the calculation of relative ion abundance. It is important to note that this approach deviates from EURL guidance requirements, and for definitive confirmation, an additional orthogonal separation or alternative detection technique must be employed.

In the current study, the inclusion of PFOSA, a neutral PFAS, needs some extra clarification. As a neutral compound, PFOSA does not interact with the anion exchange mechanism of the SPE cleanup procedure, only interacting with the backbone material based on its hydrophobicity. During the SPE procedure, the cartridge is flushed with methanol, causing a large fraction of PFOSA to elute from the column. Only a small part is eluted in the final elution step. This fraction is sufficient for the quantitative determination of PFOSA, but due to the lower absolute recovery, only with a higher detection limit and a larger variance in recovery. The PFOSA recovery can be improved by collecting, evaporating, reconstituting, and injecting the methanolic wash fraction separately.

Additionally, another challenging compound to analyze is HFPO-DA, known for its susceptibility to degradation under specific conditions. To test for the degradation of HFPO-DA, a single-factor ANOVA was conducted on the relative standard deviation of the signals of the internal standards for HFPO-DA, PFOA and PFOS. No significant variance was observed in the signal of the internal standard of HFPO-DA compared to PFOA and PFOS (*p* = 0.39, among all matrix categories). Consequently, the null hypothesis was rejected, suggesting that any potential degradation of HFPO-DA is negligible during the evaporation of the extracts. This ANOVA analysis was based on a total of 10 individual measurements, with all matrix categories included twice.

### Validation

The determined LOQs for each matrix category are presented in Table 3. We selected the definition of the LOQ fitting the aim of this research: exposure assessment. A clear definition of the LOQ is crucial to obtain reliable data as requested by the risk assessors. Unfortunately, the definition of the LOQ and the determination of it is not harmonized. This commonly results in underestimations of the actual LOQ, since often system-LOQs are used, instead of method-LOQs. This often results in potential false positives and an overestimated risk.^33^

**Table 3:** Determined LOQs Per Matrix Category (ng/kg)^a^

analyte	leafy vegetables	bulb vegetables and leek	root vegetables	fruit	other vegetables
PFPeA	25	10	100	100	25
PFHxA	1.0	1.0	0.5	2.5	1.0
PFHpA	0.5	1.0	2.5	2.5	0.5
PFOA	25^b^	25^b^	10^b^	25^b^	25^b^
PFNA	0.5	2.5^b^	1.0	1.0	0.5
PFDA	0.5	2.5^b^	0.5	0.5	0.5
PFUnDA	0.5	2.5^b^	2.5^b^	0.5	0.5
PFDoDA	0.5	1.0^b^	2.5^c^	0.5	0.5
PFTrDA^d^					
PFTeDA	500	1	100	2.5	500
PFBS	0.5	0.5	0.5	0.5	0.5
PFHxS	0.5	0.5	0.5	1.0	0.5
PFHpS	0.5	0.5	0.5	0.5	0.5
PFOS	0.5	1	0.5	0.5	0.5
PFDS	1.0	1.0	1.0	1.0	1.0
PFOSA	25	2.5	2.5	0.5	2.5
HFPO–DA	0.5	2.5	2.5	2.5	1.0
DONA	1.0	2.5	2.5	5.0	0.5
9Cl-PF3ONS	0.5	0.5	0.5	0.5	0.5
11Cl-PF3OUdS	0.5	0.5	0.5	0.5	0.5

a LOQs determined on the basis of a signal in the procedural blank or calibrants’ matrix are indicated with an asterisk.

b The LOQ was determined by multiplying the PFAS concentration in the procedural blank by a factor of 3.3.

c The LOQ was increased due to a small blank contamination of the calibration curve.

d PFTrDA did not meet the quantitative performance criteria at all levels and, as such, PFTrDA can only be analyzed qualitatively using this method.

The apparent recoveries and RSD_r_’s were first calculated within each matrix category. Upon comparing the outcomes across different categories, no statistically significant differences were observed. As a result, it was decided to combine all matrix groups to determine the method performance characteristics. Note that in all series, the MFS calibration was based on a matrix from the same category as the actual samples. As such, this is also applied in the practical application of the method. The validation results for apparent recovery, RSD_r_, and RSD_RL_ at all the validation levels are presented in Table 4.

**Table 4:** Validation Results at the Validation Concentration Levels

analyte	spike level (ng kg^–1^)	number of samples confirmed^a^	apparent recovery (%)	RSD_r_ (%)	RSD_RL_ (%)	conclusion
PFPeA	500	29	97	13	17	quan
PFHxA	2.5	29	95	15	16	quan
	50	30	102	4	5	
	500	30	103	3	3	
PFHpA	2.5	19^b^	95	12	13	quan
	50	30	100	10	11	
	500	30	103	4	4	
PFOA	50	29	103	4	8	quan
	500	30	99	7	8	
PFNA	2.5	30	97	9	11	quan
	50	30	103	4	8	
	500	30	101	4	7	
PFDA	2.5	30	101	12	12	quan
	50	30	107	9	10	
	500	30	101	7	7	
PFUnDA	2.5	30	95	16	16	quan
	50	30	102	6	7	
	500	30	102	4	5	
PFDoDA	2.5	29	113	23	23	quan
	50	30	103	5	6	
	500	30	102	4	5	
PFTrDA	2.5	14	146	35	44	qual
	50	30	134	63	64	
	500	30	139	52	57	
PFTeDA	500	30	108	8	10	quan
PFBS	2.5	29	97	21	24	quan
	50	30	102	5	6	
	500	30	103	4	5	
PFHxS	2.5	29	104	9	9	quan
	50	30	106	6	7	
	500	30	107	3	6	
PFHpS	2.5	30	105	16	17	quan
	50	30	104	12	15	
	500	30	105	12	13	
PFOS	2.5	28^b^	104	13	14	quan
	50	30	101	4	5	
	500	30	104	3	4	
PFDS	2.5	30	94	16	25	quan
	50	30	90	13	23	
	500	30	92	13	24	
PFOSA	50	30	101	7	10	quan
	500	30	102	6	7	
HFPO–DA	2.5	23^c^	96	17	17	quan
	50	24	108	6	7	
	500	30	107	8	9	
DONA	50	30	119	17	21	quan
	500	30	103	13	20	
9Cl-PF3ONS	2.5	30	105	23	23	quan
	50	30	101	24	23	
	500	30	103	22	22	
11Cl-PF3OUdS	2.5	30	99	27	37	qual
	50	30	94	21	28	quan
	500	30	97	18	28	

a Samples complying with the confirmatory criteria as described in “Confirmation of Peak Identity”.

b Rejected samples demonstrated to contain the specific PFAS. In these cases, the addition of 2.5 ng/kg did not result in a substantial signal increase. Therefore, no quantitative data at this concentration could be obtained.

c HFPO–DA showed to have a severe interference in some ion transitions mainly in onions.

The method proved to be fit-for-purpose for quantification and confirmation of most PFASs included in all matrix categories. PFTrDA did not meet the quantitative performance criteria at all levels and as such, PFTrDA can only be analyzed qualitatively using this method. This is a direct result of the absence of a fitting internal standard. Also for PFDS, DONA, 9Cl-PF3ONS, and 11Cl-PF3OUdS no isotopically labeled internal standards are available. The RSD_RL_ for these substances is higher compared to the other PFASs, but they do mostly comply with the performance criteria.

The required LOQs stated by the EURL guidelines^3^ for the analysis of the EFSA-4 PFAS in fruit and vegetables are achieved for PFNA, PFHxS, and PFOS, but not for PFOA. The targeted LOQs stated by the guidelines (which are equal to the required LOQs by the commission recommendation 2022/1431) are achieved for PFNA in almost all matrix categories, PFHxS and PFOS. They were not achieved for PFNA in the category “bulb vegetables and leek” and for PFOA in all matrix categories. In all these cases the elevated LOQs are a result of a signal in the procedural blank. For PFOA this blank contribution was around 5 ng/kg in all cases and for PFNA this was approximately 0.5 ng/kg. Clearly, to achieve the target LOQs extra effort is required to eliminate the background contamination for PFOA and to a lesser extent for PFNA. That requires an extremely controlled working environment and an extreme level of quality control on solvents and consumables.

High LOQs were observed for PFPeA, indicating that the current method is unsuitable for the quantitative analysis of PFPeA at low ppts levels, as evident from the validation results. This issue is a result of background signals in the chromatogram. Most likely originating from an interfering substance that shares the same ion transition and retention time as PFPeA.^38^ Further work is needed to identify the exact cause of these elevated LOQs.

For HFPO–DA the validation of all matrix categories except “bulb vegetables and leek” complied with all quantitative and confirmative performance criteria. Only in “bulb vegetables and leek”, HFPO–DA showed high interfering signals in the ion transition used for confirmatory analysis. Furthermore, also the most abundant ion transition showed high signals. As the confirmatory criteria were not met, it cannot be stated if HFPO–DA is present in these samples at a high level or if another substance is interfering with the quantification and confirmation of HFPO–DA.

Some compounds showed a higher variability in the LOQ between matrix categories. PFTeDA’s LOQs ranged from 500 pg/g in leafy greens and other vegetables to as low as 1 pg/g in bulb vegetables. The variability may be caused by the low absolute recovery of PFTeDA, mainly attributed to its tendency to adsorb to the LC-vial. For some matrices PFTeDA remained better in solution, yielding lower LOQs for 3 of the 5 validated categories (Table 3). Future work will be undertaken to improve the solubility of PFTeDA and other long-chain compounds, to improve the absolute recovery.

### Application

The developed method was applied to analyze of 215 fruit and vegetable samples obtained from Dutch grocery stores and weekly markets, including 35 leaf vegetables, 23 bulb vegetables including leeks, 25 root vegetables, 50 fruit, and 82 other vegetables. Note that, in specific series, lower or slightly higher LOQs were achieved compared to the validation due to a lower signal in the procedural blanks.

**Figure 2:**
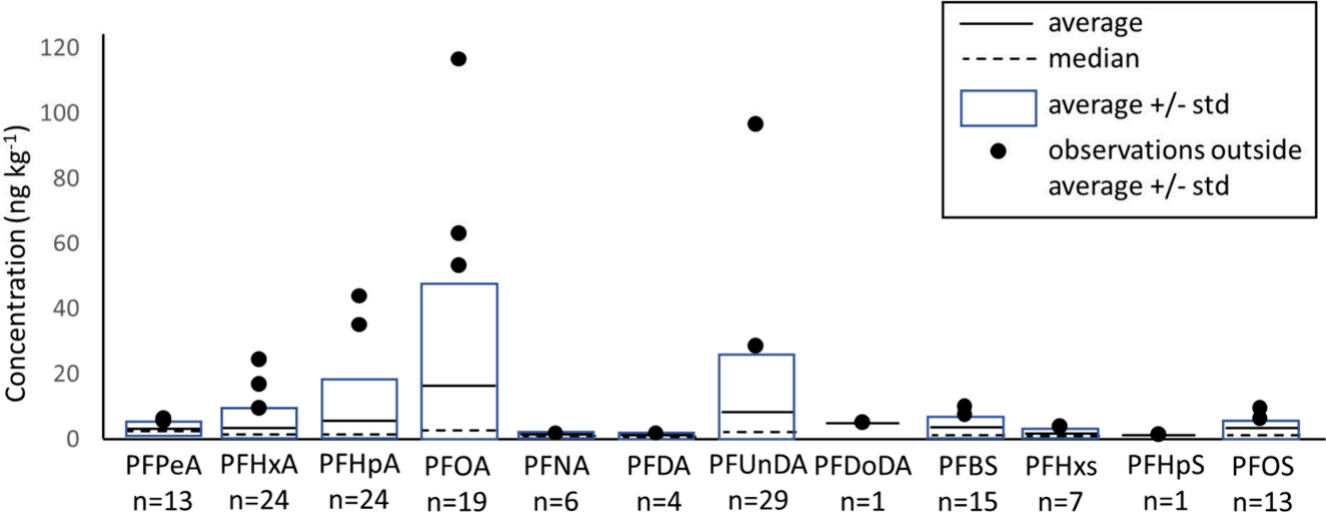
Schematic representation of detected PFAS concentrations in the fruit and vegetable samples, per PFAS. Detected PFASs are individual observations, with no sum-concentrations of different samples. *n* = number of occasions that a specific PFAS was detected in the samples (number of samples = 215).

Out of the 215 fruit and vegetables, the presence of one or more PFASs was confirmed in 87 (40.5%) samples. These included 25 leaf vegetables (71%), 3 bulb vegetables and leek (13%), 20 root vegetables (80%), 21 fruit (42%), and 18 other vegetables (22%). It is common to detect multiple PFASs in a single sample, with a total of 156 PFASs confirmed, reaching a maximum of 7 in a single sample. Concentrations ranged from 0.3 ng/kg to 117 ng/kg, indicating a highly right-skewed distribution. The monitoring data can be found in the Risk assessment of exposure to PFAS through food and drinking water by the RIVM.^39^ A schematic presentation of the results is shown in Figure 2.

**Figure 3:**
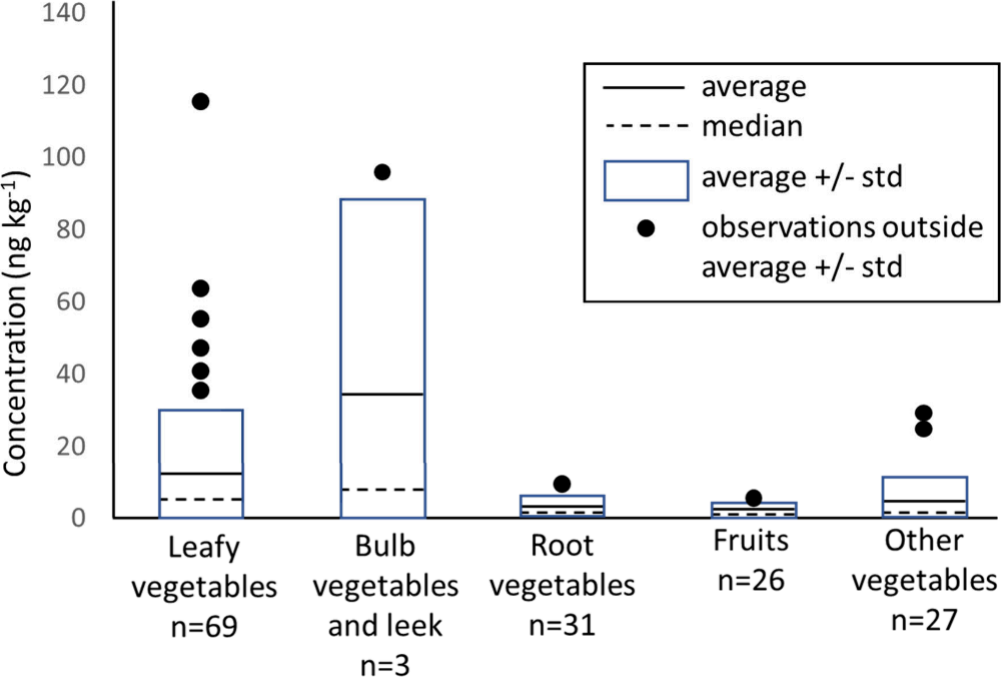
Schematic representation of detected PFAS concentrations in the fruit and vegetable samples, per matrix category. Detected PFASs are individual observations, no sum-concentrations of different PFASs. *n* = number of occasions that a PFAS was detected in the samples (number of samples = 215).

Root vegetables have the highest frequency of PFAS detection (80%), but concentrations are all below 7 ng/kg. Mainly PFPeA and PFBS were detected. Leafy vegetables also have a high frequency of contamination (71%) and in this category, the highest concentration was found, mainly of PFOA followed by PFHpA and PFHxA. The highest concentrations were found in crisp lettuce, followed by endives and spinach. Fruit has a lower frequency of occurrence of PFASs (42%) with no specific type of fruit standing out: mainly PFUnDA and PFOA were found, all at concentrations below 6 ng/kg. Other vegetables have a frequency of detection of 22%. In specific cases elevated concentrations were detected, in all cases for PFUnDA. The category “bulb vegetable and leek” seems to have relatively high PFAS content, see Figure 3. However, the frequency of detection is low, and only in one case an elevated concentration was found in a leek sample: 96 ng/kg PFUnDA.

Interestingly, the data suggest a relation between the matrix category and the PFASs detected. PFPeA was mainly found in the root vegetables. PFHpA, PFNA, and PFBS were most prominent in leafy vegetables. PFOA was only found in fruit, leafy vegetables, and root vegetables, not in the other two categories. More generic, the above-ground vegetables and fruit seem to contain mainly C_7_ – C_11_ carboxylic acids and some PFOS, whereas the underground vegetables contain mainly the shorter chain carboxylic acids and sulfonates: PFPeA and PFBS. Most likely, the observed effects are the result of matrix-specific uptake kinetics and are also influenced by different exposure routes, e.g. via uptake from soil and direct contact with irrigation/sprinkling water and air. For the latter two, the PFAS concentration is related to the plant surface area to mass ratio.

In general, the observations are in good agreement with the data reported previously. It was demonstrated that in Belgium, most similar to The Netherlands, PFOA contamination mainly occurs in leafy vegetables and root vegetables.^12^ Also, the concentration levels for the EFSA-4 PFASs are in good agreement. Also^10^ demonstrated high accumulation of PFOA in leafy vegetables and grapes. Furthermore, the finding of PFOA and PFOS in carrots and the finding of a series of PFCAs in lettuce is in agreement with previously published data.^14^ The finding of multiple PFCAs in potato as previously reported^14^ is not in agreement with the current study, where only mainly PFPeA was detected in potatoes.

According to multiple publications,^9,13,20^ in fruit and vegetables most often PFBA was detected. Furthermore, in uptake studies^22,23^ it was reported that mainly the short-chain PFASs are taken up by leafy vegetables and crops. Unfortunately, in the current study, PFBA could not be determined according to current quality standards. Notably, we observed higher concentrations of PFHxA and longer chains compared to PFPeA in all positive samples except potatoes. Uptake kinetics could be different among fruit and vegetable species. Another explanation for the observed difference could be the occurrence of different exposure routes and spatial effects (e.g., related to PFAS use and the occurrence of PFAS hotspots in the vicinity of the production site). Note the potential lack of selectivity for PFBA as previously mentioned.

Among the PFASs detected, the finding of PFUnDA stands out: it is found more often than expected and at higher levels: PFUnDA has not been previously reported and also no applications of PFUnDA are known. Even though it is unknown what the origin of PFUnDA is, its presence was confirmed by the observation of two ion transitions, a correct relative ion abundance, and a relative retention time.

As most of the concentration levels of PFAS in fruit and vegetables are low, it is important to develop and apply analytical methods with low LOQs when studying human exposure to PFASs through consumption of fruit and vegetable consumption. The method proved to be useful in detecting the currently deemed most relevant PFASs and important analogs, at relevant levels. The LOQs of some of the PFASs should be lowered further. However, these challenges arise primarily due to background signals originating from laboratory consumables, solvents, and the working environment. Special requirements may therefore be needed to further lower the LOQs.

## Supporting Information Available

Mass transitions and collision settings (SI-1), sampling scheme and validation design (SI-2), MRM chromatograms for PFHxS, PFOS, PFOA, and PFNA spiked at 1 ng/kg in an apple sample (SI-3), and list of samples and their land of origin (SI-4) (PDF)

## References

[ref1] Buck R. C.; Franklin J.; Berger U.; Conder J. M.; Cousins I. T.; De Voogt P.; Jensen A. A.; Kannan K.; Mabury S. A.; Van Leeuwen S. P. Perfluoroalkyl and polyfluoroalkyl substances in the environment: Terminology, classification, and origins. Integr. Environ. Assess. Manage. 2011, 7 (4), 513–541. 10.1002/ieam.258. PMC3214619 21793199

[ref2] Schrenk D.; Bignami M.; Bodin L.; Chipman J. K.; del Mazo J.; Grasl-Kraupp B.; Hogstrand C.; Hoogenboom L. R.; Leblanc J.-C.; Nebbia C. S.; Nielsen E.; Ntzani E.; Petersen A.; Sand S.; Vleminckx C.; Wallace H.; Barregård L.; Ceccatelli S.; Cravedi J.-P.; Halldorsson T. I.; Haug L. S.; Johansson N.; Knutsen H. K.; Rose M.; Roudot A.-C.; Van Loveren H.; Vollmer G.; Mackay K.; Riolo F.; Schwerdtle T. Risk to human health related to the presence of perfluoroalkyl substances in food. EFSA J. 2020, 18 (9), 6223 10.2903/j.efsa.2020.6223. PMC7507523 32994824

[ref3] European Union Reference Laboratory for Halogenated Persistent Organic Pollutants in Feed and Food (EURL-POPs). Guidance Document on Analytical Parameters for the Determination of Per- and Polyfluoroalkyl Substances (PFAS) in Food and Feed; EURL-POPs: Baden-Wuerttemberg, Germany, 2022; https://eurl-pops.eu/news/guidance-document-pfas/guidance-document-pfas.

[ref4] Commission Recommendation (EU) 2022/1431 of 24 August 2022 on the monitoring of perfluoroalkyl substances in food. Off. J. Eur. Union 2022, 221, 105–109.

[ref5] Jiménez-Skrzypek G.; González-Sálamo J.; Hernández-Borges J. Analytical methodologies and occurrence of per- and polyfluorinated alkyl substances—A review. J. Chromatogr. Open 2023, 4, 100089 10.1016/j.jcoa.2023.100089.

[ref6] Pasecnaja E.; Bartkevics V.; Zacs D. Occurrence of selected per- and polyfluorinated alkyl substances (PFASs) in food available on the European market—A review on levels and human exposure assessment. Chemosphere 2022, 287, 132378 10.1016/j.chemosphere.2021.132378. 34592212

[ref7] Meng P. P.; DeStefano N. J.; Knappe D. R. U. Extraction and Matrix Cleanup Method for Analyzing Novel Per- and Polyfluoroalkyl Ether Acids and Other Per- and Polyfluoroalkyl Substances in Fruits and Vegetables. J. Agric. Food Chem. 2022, 70 (16), 4792–4804. 10.1021/acs.jafc.1c07665. 35188387

[ref8] Cornelis C.; D’Hollander W.; Roosens L.; Covaci A.; Smolders R.; Van Den Heuvel R.; Govarts E.; Van Campenhout K.; Reynders H.; Bervoets L. First assessment of population exposure to perfluorinated compounds in Flanders, Belgium. Chemosphere 2012, 86 (3), 308–314. 10.1016/j.chemosphere.2011.10.034. 22104337

[ref9] Li N. K.; Song X. C.; Shen P.; Zhao C. Rapid Determination of Perfluoroalkyl and Polyfluoroalkyl Substances (PFASs) in Vegetables by on-Line Solid-Phase Extraction (SPE) with Ultra-High-Performance Liquid Chromatography–Tandem Mass Spectrometry (UHPLC–MS/MS). Anal. Lett. 2022, 55 (14), 2227–2238. 10.1080/00032719.2022.2051044.

[ref10] Li P. Y.; Oyang X. H.; Zhao Y. L.; Tu T. Q.; Tian X. J.; Li L.; Zhao Y.; Li J. Y.; Xiao Z. Y. Occurrence of perfluorinated compounds in agricultural environment, vegetables, and fruits in regions influenced by a fluorine-chemical industrial park in China. Chemosphere 2019, 225, 659–667. 10.1016/j.chemosphere.2019.03.045. 30903841

[ref11] Rawn D. F. K.; Menard C.; Feng S. Y. Method development and evaluation for the determination of perfluoroalkyl and polyfluoroalkyl substances in multiple food matrices. Food Addit. Contam.: Part A 2022, 39 (4), 752–776. 10.1080/19440049.2021.2020913. 35119964

[ref12] Herzke D.; Huber S.; Bervoets L.; D’Hollander W.; Hajslova J.; Pulkrabova J.; Brambilla G.; De Filippis S. P.; Klenow S.; Heinemeyer G.; de Voogt P. Perfluorinated alkylated substances in vegetables collected in four European countries; occurrence and human exposure estimations. Environ. Sci. Pollut. Res. 2013, 20 (11), 7930–7939. 10.1007/s11356-013-1777-8. 23686789

[ref13] Sznajder-Katarzynska K.; Surma M.; Cieslik E.; Wiczkowski W. The perfluoroalkyl substances (PFASs) contamination of fruits and vegetables. Food Addit. Contam.: Part A 2018, 35 (9), 1776–1786. 10.1080/19440049.2018.1502477. 30036163

[ref14] Haug L. S.; Salihovic S.; Jogsten I. E.; Thomsen C.; van Bavel B.; Lindström G.; Becher G. Levels in food and beverages and daily intake of perfluorinated compounds in Norway. Chemosphere 2010, 80 (10), 1137–1143. 10.1016/j.chemosphere.2010.06.023. 20599247

[ref15] Scordo C. V. A.; Checchini L.; Renai L.; Orlandini S.; Bruzzoniti M. C.; Fibbi D.; Mandi L.; Ouazzani N.; Del Bubba M. Optimization and validation of a method based on QuEChERS extraction and liquid chromatographic-tandem mass spectrometric analysis for the determination of perfluoroalkyl acids in strawberry and olive fruits, as model crops with different matrix characteristics. J. Chromatogr. A 2020, 1621, 461038 10.1016/j.chroma.2020.461038. 32199674

[ref16] Domingo J. L.; Jogsten I. E.; Eriksson U.; Martorell I.; Perelló G.; Nadal M.; van Bavel B. Human dietary exposure to perfluoroalkyl substances in Catalonia, Spain. Temporal trend. Food Chem. 2012, 135 (3), 1575–1582. 10.1016/j.foodchem.2012.06.054. 22953896

[ref17] Vestergren R.; Berger U.; Glynn A.; Cousins I. T. Dietary exposure to perfluoroalkyl acids for the Swedish population in 1999, 2005 and 2010. Environ. Int. 2012, 49, 120–127. 10.1016/j.envint.2012.08.016. 23018201

[ref18] D’Hollander W.; Herzke D.; Huber S.; Hajslova J.; Pulkrabova J.; Brambilla G.; De Filippis S. P.; Bervoets L.; de Voogt P. Occurrence of perfluorinated alkylated substances in cereals, salt, sweets and fruit items collected in four European countries. Chemosphere 2015, 129, 179–185. 10.1016/j.chemosphere.2014.10.011. 25455675

[ref19] Sungur S.; Koroglu M.; Turgut F. Determination of perfluorooctanoic acid (PFOA) and perfluorooctane sulfonic acid (PFOS) in food and beverages. Int. J. Environ. Anal. Chem. 2018, 98 (4), 360–368. 10.1080/03067319.2018.1468440.

[ref20] Noorlander C. W.; van Leeuwen S. P. J.; te Biesebeek J. D.; Mengelers M. J. B.; Zeilmaker M. J. Levels of Perfluorinated Compounds in Food and Dietary Intake of PFOS and PFOA in The Netherlands. J. Agric. Food Chem. 2011, 59 (13), 7496–7505. 10.1021/jf104943p. 21591675

[ref21] Bao J.; Li C. L.; Liu Y.; Wang X.; Yu W. J.; Liu Z. Q.; Shao L. X.; Jin Y. H. Bioaccumulation of perfluoroalkyl substances in greenhouse vegetables with long-term groundwater irrigation near fluorochemical plants in Fuxin, China. Environ. Res. 2020, 188, 109751 10.1016/j.envres.2020.109751. 32531525

[ref22] Ghisi R.; Vamerali T.; Manzetti S. Accumulation of perfluorinated alkyl substances (PFAS) in agricultural plants: A review. Environ. Res. 2019, 169, 326–341. 10.1016/j.envres.2018.10.023. 30502744

[ref23] Scher D. P.; Kelly J. E.; Huset C. A.; Barry K. M.; Hoffbeck R. W.; Yingling V. L.; Messing R. B. Occurrence of perfluoroalkyl substances (PFAS) in garden produce at homes with a history of PFAS-contaminated drinking water. Chemosphere 2018, 196, 548–555. 10.1016/j.chemosphere.2017.12.179. 29329087

[ref24] Zhou Y. R.; Lian Y. J.; Sun X.; Fu L.; Duan S. R.; Shang C. F.; Jia X. X.; Wu Y. N.; Wang M. L. Determination of 20 perfluoroalkyl substances in greenhouse vegetables with a modified one-step pretreatment approach coupled with ultra performance liquid chromatography tandem mass spectrometry (UPLC–MS–MS). Chemosphere 2019, 227, 470–479. 10.1016/j.chemosphere.2019.04.034. 31003132

[ref25] Nassazzi W.; Lai F. Y.; Ahrens L. A novel method for extraction, clean-up and analysis of per- and polyfluoroalkyl substances (PFAS) in different plant matrices using LC–MS/MS. J. Chromatogr. B 2022, 1212, 123514 10.1016/j.jchromb.2022.123514. 36384073

[ref26] Piva E.; Fais P.; Ioime P.; Forcato M.; Viel G.; Cecchetto G.; Pascali J. P. Per- and polyfluoroalkyl substances (PFAS) presence in food: Comparison among fresh, frozen and ready-to-eat vegetables. Food Chem. 2023, 410, 135415 10.1016/j.foodchem.2023.135415. 36652797

[ref27] Zacs D.; Fedorenko D.; Pasecnaja E.; Bartkevics V. Application of nano-LC-nano-ESI-Orbitrap-MS for trace determination of four priority PFAS in food products considering recently established tolerable weekly intake (TWI) limits. Anal. Chim. Acta 2023, 1251, 341027 10.1016/j.aca.2023.341027. 36925299

[ref28] Benskin J. P.; Bataineh M.; Martin J. W. Simultaneous characterization of perfluoroalkyl carboxylate, sulfonate, and sulfonamide isomers by liquid chromatography-tandem mass spectrometry. Anal. Chem. 2007, 79 (17), 6455–6464. 10.1021/ac070802d. 17665875

[ref29] Sadia M.; Yeung L. W. Y.; Fiedler H. Trace level analyses of selected perfluoroalkyl acids in food: Method development and data generation. Environ. Pollut. 2020, 263, 113721 10.1016/j.envpol.2019.113721. 32229370

[ref30] Liberatore H. K.; Jackson S. R.; Strynar M. J.; McCord J. P. Solvent Suitability for HFPO-DA (“GenX” Parent Acid) in Toxicological Studies. Environ. Sci. Technol. Lett. 2020, 7 (7), 477–481. 10.1021/acs.estlett.0c00323. 32944590 PMC7490830

[ref31] Zhang C. H.; McElroy A. C.; Liberatore H. K.; Alexander N. L. M.; Knappe D. R. U. Stability of Per- and Polyfluoroalkyl Substances in Solvents Relevant to Environmental and Toxicological Analysis. Environ. Sci. Technol. 2022, 56 (10), 6103–6112. 10.1021/acs.est.1c03979. 34734715 PMC9065217

[ref32] Berendsen B. J. A.; Lakraoui F.; Leenders L.; van Leeuwen S. P. J. The analysis of perfluoroalkyl substances at ppt level in milk and egg using UHPLC–MS/MS. Food Addit. Contam.: Part A 2020, 37 (10), 1707–1718. 10.1080/19440049.2020.1794053. 32717169

[ref33] Delatour T.; Mottier P.; Gremaud E. Limits of suspicion, recognition and confirmation as concepts that account for the confirmation transitions at the detection limit for quantification by liquid chromatography-tandem mass spectrometry. J. Chromatogr. A 2007, 1169 (1–2), 103–110. 10.1016/j.chroma.2007.08.065. 17880986

[ref34] Wenzl T.; Haedrich J.; Schaechtele A.; Piotr R.; Stroka J.; Eppe G.; Scholl G. Guidance Document on the Estimation of LOD and LOQ for Measurements in the Field of Contaminants in Food and Feed; Institute for Reference Materials and Measurements (IRMM): Geel, Belgium, 2016.

[ref35] Geosyntec Consultants. Corrective Action Plan Appendix C: K_ow_, K_oc_ and Mass Distribution Calculations; Geosyntec Consultants: Boca Raton, FL, 2019; TR0795.

[ref36] Zenobio J. E.; Salawu O. A.; Han Z. W.; Adeleye A. S. Adsorption of per- and polyfluoroalkyl substances (PFAS) to containers. J. Hazard. Mater. Adv. 2022, 7, 100130 10.1016/j.hazadv.2022.100130.

[ref37] Eun H.; Yamazaki E.; Taniyasu S.; Miecznikowska A.; Falandysz J.; Yamashita N. Evaluation of perfluoroalkyl substances in field-cultivated vegetables. Chemosphere 2020, 239, 124750 10.1016/j.chemosphere.2019.124750. 31526995

[ref38] Bangma J.; McCord J.; Giffard N.; Buckman K.; Petali J.; Chen C. L.; Amparo D.; Turpin B.; Morrison G.; Strynar M. Analytical method interferences for perfluoropentanoic acid (PFPeA) and perfluorobutanoic acid (PFBA) in biological and environmental samples. Chemosphere 2023, 315, 137722 10.1016/j.chemosphere.2022.137722. 36592832 PMC10165721

[ref39] Schepens M. A. A.; te Biesebeek J. D.; Hartmann J.; van der Aa N. G. F. M.; Zijlstra R.; Boon P. E. Risk assessment of exposure to PFAS through food and drinking water in the Netherlands (Risicobeoordeling van blootstelling aan PFAS via voedsel en drinkwater in Nederland); Rijksinstituut voor Volksgezondheid en Milieu (RIVM): Bilthoven, Netherlands, 2023.

