# Supplementary material for: Development and Application of a Liquid Chromatography–Tandem Mass Spectrometry Method for the Analysis of 20 Perfluoroalkyl Substances in Fruit and Vegetables at Sub-Parts-per-Trillion Levels
Source: J Agric Food Chem. 2024 Aug 7;72(33):18731–41. doi: 10.1021/acs.jafc.4c01172 (PMC11342373; doi:10.1021/acs.jafc.4c01172)
Supplement: Supplementary file 1 — jf4c01172_si_001.pdf [file jf4c01172_si_001.pdf]

Supporting Information

Development and Application of an LC-MS/MS Method for the Analysis of 20 Perfluoroalkyl Substances in Fruit and Vegetables at Sub-parts-per-Trillion Levels

Ruben Kause[a]*, Stefan van Leeuwen[a], Kerstin Krätschmer[a], Bob van Dooren[a], Rens Keppels[a], Helgah Makarem[a], L. Ron A.P. Hoogenboom[a], Leontien de Pagter-de Witte[a], and Bjorn J.A. Berendsen[a]

[a] Wageningen Food Safety Research (WFSR), Wageningen University & Research, 6708 WB Wageningen, The Netherlands

*Email: ruben.kause@wur.nl;

Phone: (+31) 317 489 608

Contents

SI-1 Mass transitions and collision-settings 2

SI-2 Sampling scheme and validation design 4

SI-3 MRM-chromatograms for PFHxS, PFOS, PFOA and PFNA spiked at 1 ng/kg in an apple sample 5

SI-4 List of samples and their land of origin 8

SI-1 Mass transitions and collision-settings

Table S1: Mass transitions of quantifier, qualifier and labelled internal standard for each PFAS included in the LC-ESI-MS/MS method. Compounds marked with an asterisk are quantified using a different internal standard.

Compound	Type	Q1 [m/z]	Q3 [m/z]	CE [V]	CXP [V]	RT [min]
Perfluoropentanoic acid (PFPeA)	T1	263.0	219.0	-12	-8	5.85
	T2	263.0	263.0	-5	-8	5.85
IS = $^{13}C_3$-PFPeA	IS	266.0	222.0	-12	-16	5.85
Perfluorohexanoic acid (PFHxA)	T1	313.0	269.0	-14	-19	7.25
	T2	313.0	119.0	-26	-7	7.25
IS = $^{13}C_2$-PFHxA	IS	315.0	270.0	-14	-17	7.25
Perfluoroheptanoic acid (PFHpA)	T1	363.0	319.0	-16	-9	8.13
	T2	363.0	169.0	-25	-8	8.13
IS = $^{13}C_4$-PFpA	IS	367.0	322.0	-15	-22	8.13
Perfluorononanoic acid (PFNA)	T1	463.0	419.0	-17	-8	9.31
	T2	463.0	219.0	-24	-11	9.31
IS = $^{13}C_5$-PFNA	IS	468.0	423.0	-16	-29	9.31
Perfluorodecanoic acid (PFDA)	T1	513.0	219.0	-25	-11	9.75
	T2	513.0	469.0	-17	-10	9.75
IS = $^{13}C_2$-PFDA	IS	515.0	470.0	-17	-29	9.75
Perfluoroundecanoic acid (PFUnDA)	T1	563.0	519.0	-18	-10	10.16
	T2	563.0	269.0	-27	-16	10.16
IS = $^{13}C_2$-PFUnDA	IS	565.0	520.0	-17	-32	10.16
Perfluorododecanoic acid (PFDoDA)	T1	613.0	319.0	-28	-18	10.5
	T2	613.0	569.0	-20	-12	10.5
IS = $^{13}C_2$-PFDoDA	IS	615.0	570.0	-19	-16	10.5
Perfluorotridecanoic acid (PFTrDA*)	T1	663.0	269.0	-30	-14	10.8
	T2	663.0	619.0	-20	-15	10.8
**IS = $^{13}C_2$-PFDoDA*	*IS*	*615.0*	*570.0*	*-19*	*-16*	*10.5*
Perfluorotetradecanoic acid (PFTeDA)	T1	713.0	319.0	-32	-23	11.12
	T2	713.0	669.0	-20	-38	11.12
IS = $^{13}C_2$-PFTeDA	IS	715.0	670.0	-20	-15	11.12
Perfluorobutane sulphonic acid (PFBS)	T1	299.0	79.9	-60	-9	6.32
	T2	299.0	98.9	-37	-11	6.32
IS = $^{13}C_3$-PFBS	IS	302.0	79.9	-64	-9	6.32
Perfluorohexane sulphonic acid (PFHxS)	T1	399.0	79.9	-88	-9	8.18
	T2	399.0	98.9	-75	-11	8.18
IS = $^{18}O_2$-PFHxS	IS	403.0	83.9	-74	-9	8.18
Perfluoroheptane sulphonic acid (PFHpS*)	T1	449.0	79.9	-96	-9	8.78
	T2	449.0	98.9	-79	-11	8.78
**IS = $^{18}O_2$-PFHxS*	*IS*	*403.0*	*83.9*	*-74*	*-9*	*8.18*

Table S1: Continued

Compound	Type	Q1 [m/z]	Q3 [m/z]	CE [V]	CXP [V]	RT [min]
Perfluorooctane sulphonic acid (PFOS)	T1	499.0	79.9	-104	-9	9.29
	T2	499.0	98.9	-95	-11	9.29
IS = $^{13}C_4$-PFOS	IS	503.0	79.9	-107	-12	9.29
InjS = *$^{13}C_8$-PFOS*	InjS	507.0	79.9	-108	-11	9.29
Perfluorooctanoic acid (PFOA)	T1	413.0	169.0	-26	-9	8.78
	T2	413.0	369.0	-15	-7	8.78
IS = $^{13}C_4$-PFOA	IS	417.0	372.0	-15	-17	8.78
InjS = *$^{13}C_8$-PFOA*	InjS	421.0	376.0	-16	-25	8.78
Perfluorodecane sulphonic acid (PFDS*)	T1	599.0	79.9	-124	-9	9.8
	T2	599.0	230.0	-66	-13	9.8
**IS = $^{13}C_2$-PFUnDA*	*IS*	*565.0*	*520.0*	*-17*	*-32*	*10.16*
Perfluorooctanesulfonamide (PFOSA)	T1	498.0	77.9	-85	-9	9.96
	T2	498.0	48.0	-146	-22	9.96
IS = $^{13}C_8$-PFOSA	IS	506.0	77.9	-91	-8	9.96
hexafluoropropylene oxide-dimer acid	T1	285.0	169.0	-11	-9	7.57
(HFPO-DA (GenX))	T2	285.0	185.0	-23	-11	7.57
IS = $^{13}C_3$-HFPO-DA	IS	287.0	169.0	-17	-5	7.57
Dodecafluoro-3H-4,8-dioxanonanoic acid	T1	377.0	251.0	-17	-7	8.21
(DONA)*	T2	377.0	85.0	-41	-7	8.21
**IS = $^{18}O_2$-PFHxS*	*IS*	*403.0*	*83.9*	*-74*	*-9*	*8.18*
Dodecafluoro-3H-4,8-dioxanonanoic acid	T1	530.9	351.0	-38	-23	9.55
(9Cl-PF3ONS)*	T2	530.9	35.0	-89	-17	9.55
**IS = $^{13}C_4$-PFOS*	*IS*	*503.0*	*79.9*	*-107*	*-12*	*9.29*
Dodecafluoro-3H-4,8-dioxanonanoic acid	T1	630.9	450.9	-42	-25	10.32
(11Cl-PF3OUdS) *	T2	630.9	35.0	-99	-16	10.32
**IS = $^{13}C_2$-PFDoDA*	*IS*	*615.0*	*570.0*	*-19*	*-16*	*10.5*

Q1 = precursor ion (m/z)
Q3 = product ion (m/z)
CE = Collision energy (V)
CXP = Exit potential from collision cell (V)
RT = Retention time (min)
T1 = First ion transition
T2 = Second ion transition
IS = Internal standard
InjS = Injection standard
* = different IS than native compound

SI-2 Sampling scheme and validation design

Table S2: Detailed overview of the validation scheme

#	1: Leafy vegetables	2: Fruit	3: Root vegetables	4: Bulb vegetables	5: Other vegetables	Concentration [1)]
**P1 (MFS)**	Type: Spinach	Type: Apple	Type: Potato	Type: Onion	Type: Zucchini	MFS-reeks [2)]
**P2**	Type: Endive	Type: Strawberry	Type: Beets (peeled)	Type: Onion	Type: Cauliflower	+ 0 pg/g<br>+ 2.5 pg/g<br>+ 50 pg/g<br>+ 500 pg/g
**P3**	Type: Kale	Type: White grapes	Type: Beets (not peeled)	Type: Leek	Type: Broccoli	+ 0 pg/g<br>+ 2.5 pg/g<br>+ 50 pg/g<br>+ 500 pg/g
**P4**	Type: Iceberg lettuce	Type: Plum	Type: Carrot (peeled)	Type: Garlic	Type: Snow pees	+ 0 pg/g<br>+ 2.5 pg/g<br>+ 50 pg/g<br>+ 500 pg/g
**P5**	Type: Turkish lettuce	Type: Pear	Type: Carrot (not peeled)	Type: Red onion	Type: Rubarb	+ 0 pg/g<br>+ 2.5 pg/g<br>+ 50 pg/g<br>+ 500 pg/g
**P6**	Type: Swiss chard	Type: Red currants	Type: Potato (peeled)	Type: Scallions	Type: Pumpkin	+ 0 pg/g<br>+ 2.5 pg/g<br>+ 50 pg/g<br>+ 500 pg/g
**P7**	Type: Butterhead lettuce	Type: Apple	Type: Potato (not peeled)	Type: Chives	Type: Cucumber	+ 0 pg/g<br>+ 2.5 pg/g<br>+ 50 pg/g<br>+ 500 pg/g

SI-3 MRM-chromatograms for PFHxS, PFOS, PFOA and PFNA spiked at 1 ng/kg in an apple sample

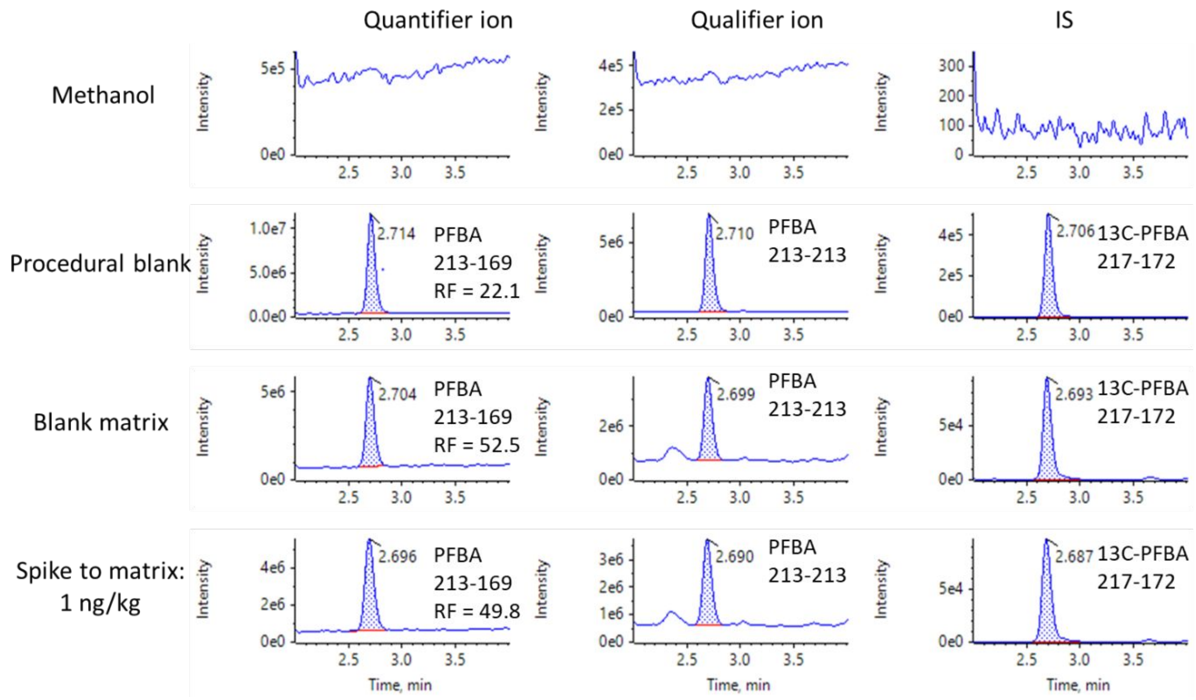

Figure S1: Top) Methanol blank (elevated background).2nd row) Procedural blank (methodologically contaminated). 3rd row) MRM chromatograms of PFBA-transitions in a blank apple sample (methodologically and naturally contaminated). Bottom) MRM chromatograms of PFBA-transitions in a fortified (1 ng/kg) apple sample (no signal increase observed, due to high contamination). Left to right: Qualifier ion, Quantifier ion, and internal standard. RF = response factor.

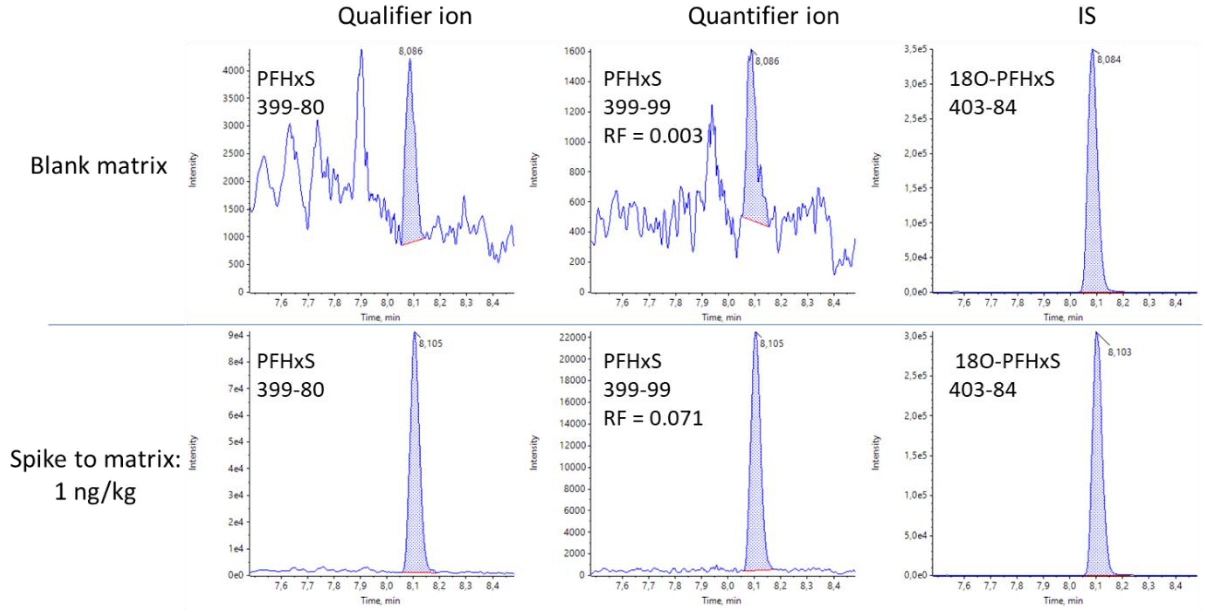

Figure S2: Top) MRM chromatograms of PFHxS-transitions in a blank apple sample (naturally contaminated). Bottom) MRM chromatograms of PFHxS-transitions in a fortified (1 ng/kg) apple sample. Left to right: Qualifier ion, Quantifier ion, and internal standard. RF = response factor.

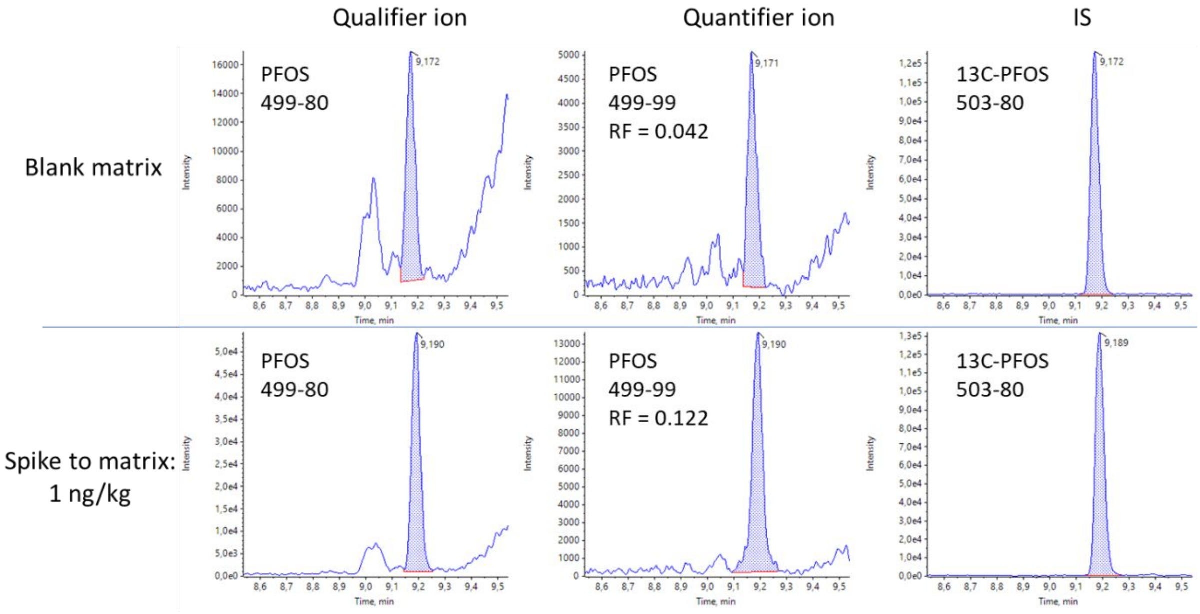

Figure S3: Top) MRM chromatograms of PFOS-transitions in a blank apple sample (naturally contaminated). Bottom) MRM chromatograms of PFOS-transitions in a fortified (1 ng/kg) apple sample. Left to right: Qualifier ion, Quantifier ion, and internal standard. RF = response factor.

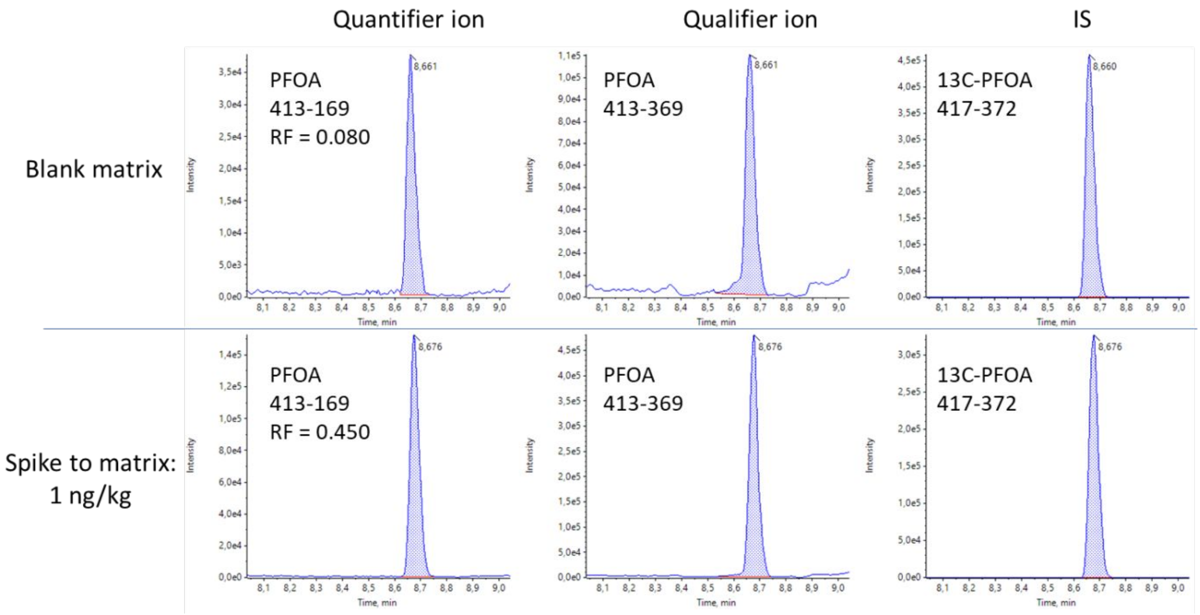

Figure S4: Top) MRM chromatograms of PFOA-transitions in a blank apple sample (naturally contaminated). Bottom) MRM chromatograms of PFOA-transitions in a fortified (1 ng/kg) apple sample. Left to right: Qualifier ion, Quantifier ion, and internal standard. RF = response factor.

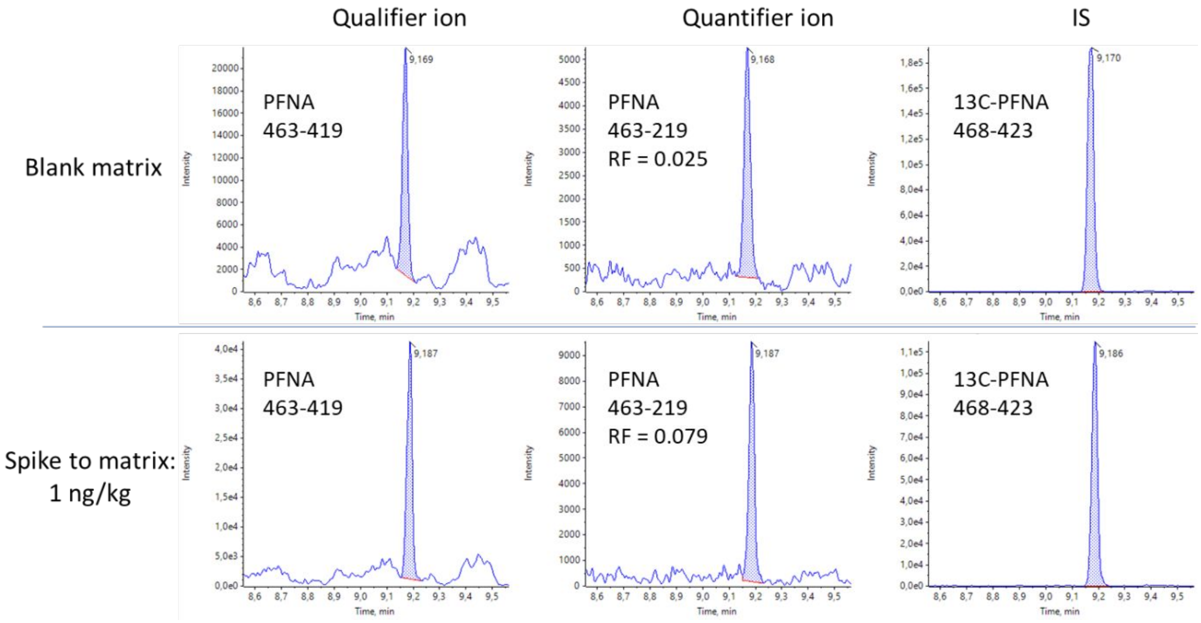

Figure S5: Top) MRM chromatograms of PFNA-transitions in a blank apple sample (naturally contaminated). Bottom) MRM chromatograms of PFNA-transitions in a fortified (1 ng/kg) apple sample. Left to right: Qualifier ion, Quantifier ion, and internal standard. RF = response factor.

SI-4 List of samples and their land of origin

Table S3: Sample-list and land of origin

#	Category	Product	Land of origin
1	Processed vegetables	Garden peas (frozen)	Peru
2	Fruits	Strawberries (including frozen)	Morocco
3	Vegetables	Spinaches (including frozen)	The Netherlands
4	Vegetables	Mushrooms	The Netherlands
5	Fruits	Strawberries (including frozen)	South Africa
6	Vegetables	Mushrooms	The Netherlands
7	Vegetables	Tomatoes and cherry tomatoes	The Netherlands
8	Fruits	Grapes	The Netherlands
9	Fruits	Grapes	The Netherlands
10	Vegetables	Tomatoes and cherry tomatoes	Spain
11	Vegetables	Potatoes	The Netherlands
12	Vegetables	Potatoes	The Netherlands
13	Fruits	Oranges	Spain
14	Vegetables	Onions	The Netherlands
15	Vegetables	Onions	The Netherlands
16	Vegetables	Carrots	The Netherlands
17	Fruits	Apples	The Netherlands
18	Fruits	Mandarins	Egypt
19	Vegetables	Belgian endives	The Netherlands
20	Vegetables	Belgian endives	The Netherlands
21	Vegetables	Sweet peppers	The Netherlands
22	Vegetables	Cucumbers	The Netherlands
23	Vegetables	Spinaches (including frozen)	Colombia
24	Vegetables	Beetroots (including vacuum packed)	Panama
25	Vegetables	Broccoli	*Unknown*
26	Vegetables	French beans	*Unknown*
27	Vegetables	Leeks	Spain
28	Vegetables	Cauliflowers	Spain
29	Vegetables	Curly endives	Spain
30	Processed vegetables	Beans, canned or jarred	The Netherlands
31	Fruits	Bananas	The Netherlands
32	Fruits	Bananas	*Unknown*
33	Processed vegetables	Sweet corn, canned	*Unknown*
34	Processed vegetables	Sweet corn, canned	Spain
35	Fruits	Strawberries (including frozen)	*Unknown*
36	Vegetables	Spinaches (including frozen)	Morocco
37	Vegetables	Mushrooms	The Netherlands
38	Vegetables	Mushrooms	The Netherlands
39	Vegetables	Leeks	The Netherlands
40	Vegetables	Cucumbers	The Netherlands
41	Vegetables	Onions	Spain
42	Vegetables	Onions	The Netherlands
43	Vegetables	Tomatoes and cherry tomatoes	The Netherlands
44	Vegetables	Beetroots (including vacuum packed)	*Unknown*
45	Vegetables	Cauliflowers	Colombia
46	Vegetables	Carrots	The Netherlands
47	Vegetables	Belgian endives	Peru
48	Vegetables	Belgian endives	Italy
49	Vegetables	French beans	The Netherlands

50	Vegetables	Spinaches (including frozen)	Ecuador
51	Vegetables	Broccoli	Spain
52	Vegetables	Potatoes	Spain
53	Vegetables	Potatoes	*Unknown*
54	Fruits	Pears	*Unknown*
55	Fruits	Bananas	*Unknown*
56	Fruits	Apples	*Unknown*
57	Fruits	Grapes	The Netherlands
58	Fruits	Grapes	*Unknown*
59	Fruits	Strawberries (including frozen)	The Netherlands
60	Fruits	Bananas	Ecuador
61	Fruits	Mandarins	Spain
62	Vegetables	Crisp lettuces	*Unknown*
63	Processed vegetables	Sweet corn, canned	*Unknown*
64	Processed vegetables	Sweet corn, canned	*Unknown*
65	Processed vegetables	Sweet corn, canned	*Unknown*
66	Processed vegetables	Beans, canned or jarred	The Netherlands
67	Processed vegetables	Peas, canned or jarred	*Unknown*
68	Vegetables	Spinaches (including frozen)	*Unknown*
69	Vegetables	Tomatoes and cherry tomatoes	Spain
70	Fruits	Strawberries (including frozen)	The Netherlands
71	Fruits	Grapes	Brazil
72	Vegetables	Potatoes	The Netherlands
73	Vegetables	Beetroots (including vacuum packed)	Spain
74	Vegetables	Curly endives	The Netherlands
75	Vegetables	Crisp lettuces	The Netherlands
76	Vegetables	Cauliflowers	The Netherlands
77	Vegetables	Cucumbers	Spain
78	Vegetables	Cucumbers	The Netherlands
79	Vegetables	Leeks	The Netherlands
80	Vegetables	Leeks	The Netherlands
81	Fruits	Bananas	Colombia
82	Vegetables	Belgian endives	The Netherlands
83	Fruits	Pears	The Netherlands
84	Vegetables	French beans	Morocco
85	Vegetables	Tomatoes and cherry tomatoes	The Netherlands
86	Processed vegetables	Sweet corn, canned	*Unknown*
87	Processed vegetables	Sweet corn, canned	*Unknown*
88	Vegetables	Sweet peppers	The Netherlands
89	Vegetables	Sweet peppers	Spain
90	Fruits	Oranges	Spain
91	Fruits	Mandarins	Spain
92	Vegetables	Onions	The Netherlands
93	Vegetables	Mushrooms	*Unknown*
94	Vegetables	Mushrooms	*Unknown*
95	Processed vegetables	Peas, canned or jarred	*Unknown*
96	Processed vegetables	Beans, canned or jarred	*Unknown*
97	Processed vegetables	Garden peas (frozen)	The Netherlands
98	Fruits	Strawberries (including frozen)	Spain
99	Fruits	Grapes	Spain
100	Vegetables	Broccoli	Spain
101	Fruits	Bananas	Morocco
102	Vegetables	Spinaches (including frozen)	Spain
103	Vegetables	Tomatoes and cherry tomatoes	Morocco
104	Vegetables	Crisp lettuces	The Netherlands
105	Vegetables	French beans	Belgium

106	Vegetables	Cucumbers	The Netherlands
107	Fruits	Strawberries (including frozen)	The Netherlands
108	Vegetables	Beetroots (including vacuum packed)	The Netherlands
109	Vegetables	Curly endives	Spain
110	Vegetables	Belgian endives	Spain
111	Fruits	Oranges	The Netherlands
112	Vegetables	Sweet peppers	The Netherlands
113	Vegetables	Leeks	The Netherlands
114	Fruits	Pears	The Netherlands
115	Fruits	Apples	The Netherlands
116	Vegetables	Onions	*Unknown*
117	Vegetables	Potatoes	*Unknown*
118	Vegetables	Mushrooms	Spain
119	Processed vegetables	Garden peas (frozen)	The Netherlands
120	Processed vegetables	Sweet corn, canned	Egypt
121	Vegetables	Tomatoes and cherry tomatoes	The Netherlands
122	Vegetables	French beans	The Netherlands
123	Vegetables	French beans	France
124	Vegetables	Curly endives	Spain
125	Vegetables	Lettuces, excluding crisp lettuces	The Netherlands
126	Vegetables	Cucumbers	Spain
127	Vegetables	Onions	The Netherlands
128	Vegetables	Belgian endives	The Netherlands
129	Vegetables	Sweet peppers	Spain
130	Vegetables	Carrots	Spain
131	Vegetables	Cauliflowers	Spain
132	Fruits	Mandarins	Colombia
133	Vegetables	Mushrooms	The Netherlands
134	Fruits	Oranges	The Netherlands
135	Vegetables	Sweet peppers	The Netherlands
136	Fruits	Bananas	The Netherlands
137	Fruits	Apples	The Netherlands
138	Fruits	Pears	The Netherlands
139	Vegetables	Tomatoes and cherry tomatoes	The Netherlands
140	Vegetables	Onions	*Unknown*
141	Vegetables	Potatoes	Brazil
142	Vegetables	Potatoes	Peru
143	Vegetables	Leeks	*Unknown*
144	Processed vegetables	Beans, canned or jarred	*Unknown*
145	Fruits	Grapes	*Unknown*
146	Fruits	Grapes	The Netherlands
147	Vegetables	Spinaches (including frozen)	The Netherlands
148	Processed vegetables	Garden peas (frozen)	Morocco
149	Fruits	Strawberries (including frozen)	Spain
150	Vegetables	Potatoes	Poland
151	Vegetables	Potatoes	*Unknown*
152	Vegetables	Spinaches (including frozen)	The Netherlands
153	Vegetables	Carrots	The Netherlands
154	Vegetables	Curly endives	Brazil
155	Vegetables	French beans	*Unknown*
156	Vegetables	Cauliflowers	The Netherlands
157	Vegetables	Belgian endives	The Netherlands
158	Vegetables	Broccoli	Spain
159	Vegetables	Mushrooms	The Netherlands
160	Vegetables	Mushrooms	Spain
161	Vegetables	Carrots	The Netherlands

162	Vegetables	Cucumbers	Colombia
163	Vegetables	Onions	The Netherlands
164	Vegetables	Onions	The Netherlands
165	Vegetables	Leeks	Spain
166	Fruits	Bananas	The Netherlands
167	Fruits	Strawberries (including frozen)	Spain
168	Vegetables	Leeks	The Netherlands
169	Vegetables	Crisp lettuces	The Netherlands
170	Vegetables	Tomatoes and cherry tomatoes	Morocco
171	Vegetables	Sweet peppers	The Netherlands
172	Vegetables	Cucumbers	Brazil
173	Vegetables	Beetroots (including vacuum packed)	Lebanon
174	Vegetables	Tomatoes and cherry tomatoes	Spain
175	Fruits	Strawberries (including frozen)	The Netherlands
176	Fruits	Grapes	The Netherlands
177	Fruits	Grapes	The Netherlands
178	Fruits	Oranges	The Netherlands
179	Fruits	Pears	*Unknown*
180	Fruits	Apples	The Netherlands
181	Fruits	Mandarins	*Unknown*
182	Fruits	Bananas	*Unknown*
183	Vegetables	Potatoes	The Netherlands
184	Vegetables	French beans	The Netherlands
185	Vegetables	Belgian endives	The Netherlands
186	Vegetables	Onions	*Unknown*
187	Vegetables	Sweet peppers	*Unknown*
188	Vegetables	Belgian endives	The Netherlands
189	Vegetables	Leeks	Spain
190	Vegetables	Broccoli	The Netherlands
191	Vegetables	Leeks	The Netherlands
192	Processed vegetables	Sweet corn, canned	The Netherlands
193	Vegetables	French beans	Senegal
194	Vegetables	Sweet peppers	The Netherlands
195	Vegetables	Spinaches (including frozen)	The Netherlands
196	Vegetables	Potatoes	The Netherlands
197	Processed vegetables	French fries, pre-baked (including frozen)	*Unknown*
198	Processed vegetables	French fries, pre-baked (including frozen)	*Unknown*
199	Vegetables	Crisp lettuces	The Netherlands
200	Vegetables	Potatoes	*Unknown*
201	Processed vegetables	French fries, pre-baked (including frozen)	*Unknown*
202	Vegetables	Potatoes	*Unknown*
203	Vegetables	Potatoes	The Netherlands
204	Vegetables	French beans	Spain
205	Vegetables	Sweet peppers	The Netherlands
206	Vegetables	Sweet peppers	The Netherlands
207	Vegetables	Cucumbers	Spain
208	Vegetables	Leeks	The Netherlands
209	Vegetables	Onions	*Unknown*
210	Fruits	Bananas	The Netherlands
211	Vegetables	Spinaches (including frozen)	Spain
212	Vegetables	Lettuces, excluding crisp lettuces	Spain
213	Vegetables	Lettuces, excluding crisp lettuces	The Netherlands
214	Vegetables	Lettuces, excluding crisp lettuces	*Unknown*
215	Vegetables	Lettuces, excluding crisp lettuces	The Netherlands
